# Occupational Brachial Artery Injury by a Foreign Body with Subsequent Soft Tissue Hematoma Superinfection

**DOI:** 10.3390/ijerph18126400

**Published:** 2021-06-13

**Authors:** Paweł Gać, Piotr Macek, Barbara Dziadkowiec, Rafał Poręba

**Affiliations:** 1 Centre for Diagnostic Imaging, 4th Military Hospital, Weigla 5, 50-981 Wroclaw, Poland; 2 Department of Hygiene, Wroclaw Medical University, Mikulicza-Radeckiego 7, 50-368 Wroclaw, Poland; 3 Department of Internal Medicine, Occupational Diseases and Hypertension, Wroclaw Medical University, Borowska 213, 50-556 Wroclaw, Poland; piotr.macek@student.umed.wroc.pl (P.M.); rafal.poreba@umed.wroc.pl (R.P.); 4 Department of Pathophysiology, Wroclaw Medical University, ul. Marcinkowskiego 1, 50-368 Wroclaw, Poland; barbara.dziadkowiec@student.umed.wroc.pl

**Keywords:** brachial artery, computed tomography angiography, hematoma, metallic foreign body, occupational injury, superinfection

## Abstract

Vascular injuries constitute a significant problem worldwide. Nearly 90% of arterial injuries concern the vessels in the limbs, of which the arteries of the lower limbs are most often damaged in military operations, while in other cases (mainly road accidents), the vessels of the upper limbs are damaged more often. In this report, the authors present a case of occupational brachial artery injury by a foreign body with subsequent soft tissue hematoma superinfection. The presented case emphasizes the importance of computed tomography angiography as a precise diagnostic tool facilitating the planning of a surgical procedure in patients with an occupational upper limb injury.

## 1. Case Study

A 32-year-old Caucasian male, with normal body mass, with no significant medical history, not taking medication and not receiving treatment for chronic diseases, was admitted to a hospital emergency department due to a gradual increase in the mobility impairment of the right upper limb, resulting from a workplace incident, i.e., an injury to the right upper limb in the region of the cubital fossa several weeks before, as well as acute pain in the area of the injury and fever on the day of admission. Upon admission, the right upper limb was slightly larger in circumference than the left upper limb, with warmer, red skin, and compulsorily positioned, with a 90° contracture in the elbow joint. Increased CRP was found in laboratory tests, otherwise without significant deviations. Due to the overall clinical condition, a decision was made to supplement the diagnostics with computed tomography of the right upper limb in the vascular option.

**Figure 1:**
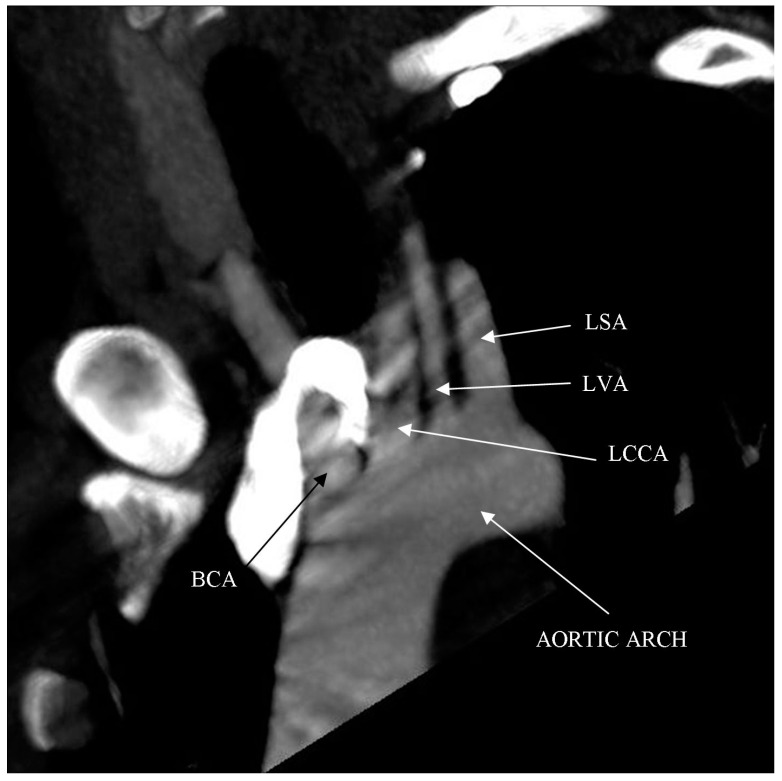
Right upper limb computed tomography angiography. MIP reconstruction. Candy cane view. Independent left vertebral artery ostium between the ostia of the left common carotid artery and the left subclavian artery (vascular anatomic variant).

**Figure 2:**
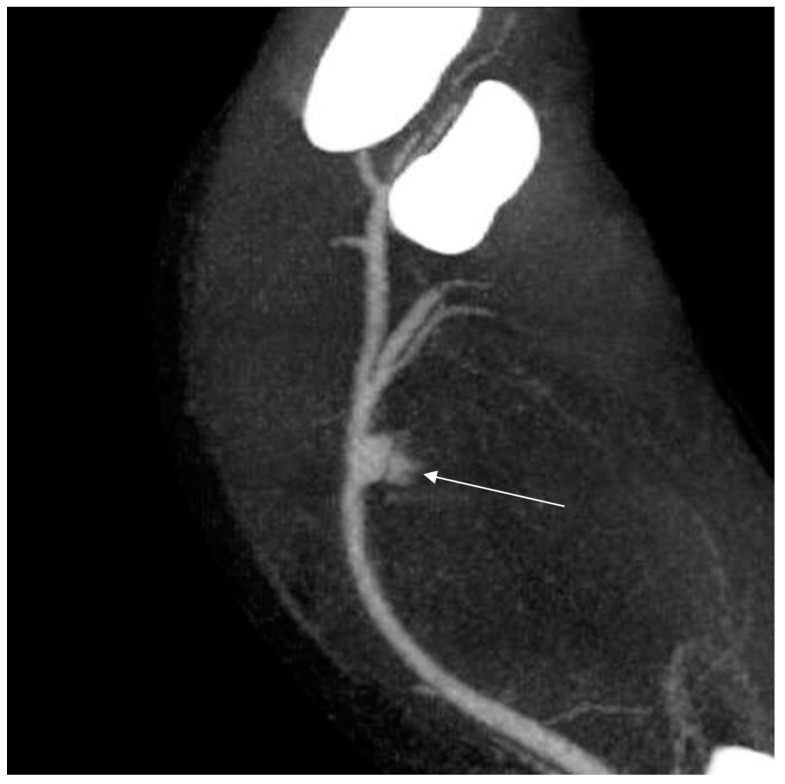
Right upper limb computed tomography angiography. MIP reconstruction. Leakage of the contrast agent beyond the lumen was found, consistent with active bleeding from the injured brachial artery.

**Figure 3:**
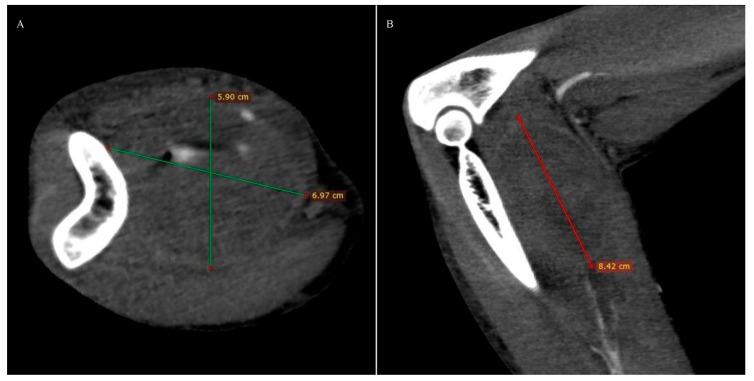
Right upper limb computed tomography angiography. MPR reconstruction. Soft tissue hematoma around the brachial artery: (**A**). Axial view. (**B**). Coronal view.

**Figure 4:**
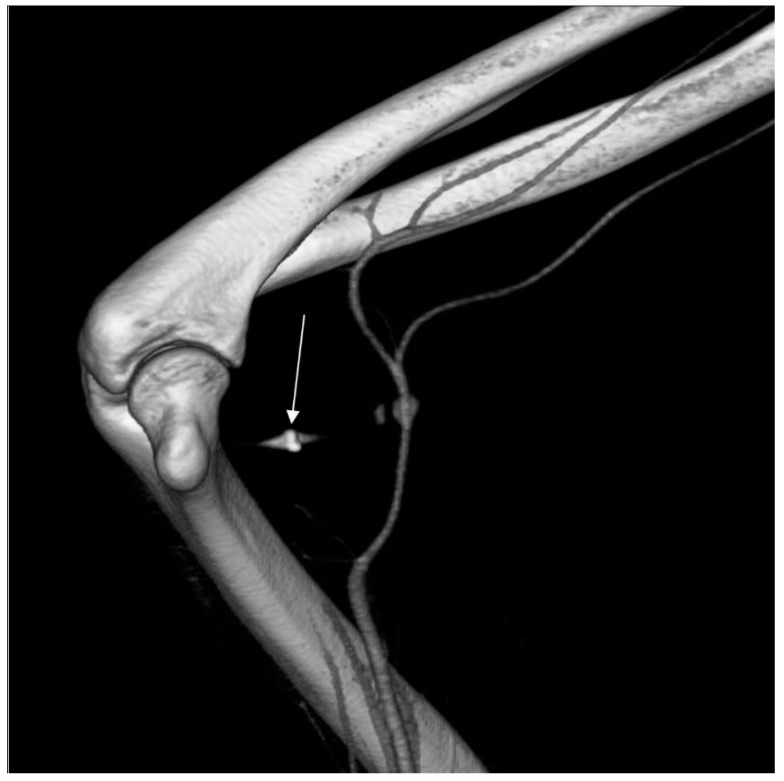
Right upper limb computed tomography angiography. VRT reconstruction. High-density, metallic foreign body within the soft tissue hematoma.

Computed tomography angiography (CTA) of the right upper limb was performed in 5.0- and 0.5-mm layers. The examination showed a non-dilated aortic arch with an independent left vertebral artery ostium between the ostia of the left common carotid artery and the left subclavian artery (Figure 1). Moreover, non-dilated arterial vessels of the right upper limb were visualized—the brachiocephalic trunk, the right subclavian artery, the right axillary artery, the right brachial artery, and the arteries of the right forearm. At the level of the distal epiphysis of the right humerus, just before the division of the brachial artery into the arteries of the forearm, a leakage of the contrast agent beyond the lumen was found, consistent with active bleeding from the injured brachial artery (Figure 2). At this level, a layered soft tissue hematoma was visualized around the brachial artery with approximate dimensions of up to 7.0 cm × 5.9 cm in cross-section and 8.4 cm in the longitudinal dimension (Figure 3). Within the soft tissue hematoma, a high-density, metallic foreign body up to about 0.7 cm in cross-sectional dimensions was localized (Figure 4).

Based on the CTA examination of the right upper limb, a decision was made to perform an urgent vascular surgery. During the procedure, the hematoma of the cubital fossa was removed together with the metallic foreign body between the clots. The damaged brachial artery was reconstructed with the use of a fragment of the basilic vein. After the procedure, limb mobility was restored, and body temperature and inflammatory parameters normalized.

## 2. Discussion

The described case concerns an upper limb injury that occurred in the workplace, with damage caused by a metallic foreign body to the brachial artery wall and subsequent formation of a soft tissue hematoma. The superinfection of the hematoma, because of the presence of a foreign body, resulted in the inflammation and exacerbation of the previously growing clinical symptoms.

Vascular injuries constitute a significant problem worldwide [1]. Nearly 90% of arterial injuries concern the vessels in the limbs, of which the arteries of the lower limbs are most often damaged in military operations, while in other cases (mainly road accidents), the vessels of the upper limbs are damaged more often. Extremity arterial injury is uncommon among civilian trauma patients, but notable for its potentially severe consequences, which include: pseudoaneurysm, arteriovenous fistulas, artery dissection, soft tissue hematoma, arterial embolism, acute limb ischemia, limb amputation, or hemorrhagic shock. Treatment is often complex due to the involvement of multiorgan system trauma, as well as concomitant local musculoskeletal injuries [2].

The importance of CTA in patients with arterial injuries was analyzed by Kelly et al. [3]. This study assessed whether CTA after physical examination changed the clinical care of patients with high energy lower limb injuries. In a group of 157 patients, it was shown that physical examination alone allows to obtain high sensitivity to vascular damage in lower limb injuries. CTA is useful for confirming and locating the source of acute vascular injury. In patients with suspected vascular injuries, a negative CTA result determines the possibility of discharge from the emergency department without further clinical observation.

Extensive tissue damage, most typical of gunshot wounds, is the optimal environment for the development of a bacterial infection. In cases of superinfected vascular injuries, it is necessary to make a tissue prosthesis after mobilizing both ends of the damaged vessel. The best method is an end-to-end anastomosis if possible. If not, a venous prosthesis can be used, because it has better indicators of patency and resistance to infections than synthetic prostheses [4]. In the study conducted by Zellweger et al., in which a three-year follow-up was carried out involving the effectiveness of treatment in 113 men and 11 women who suffered from a brachial artery injury because of a stab or gunshot wound, in approximately one-third of patients, it was possible to perform primary repair of the artery. In approximately two-thirds of patients, the use of a vein to join the ends of the damaged vessel was required [5].

Studies on the effectiveness of treatment of non-iatrogenic damage to the arteries of the limbs indicate good prognosis, assuming the performance of vascular surgery interventions. In a study conducted by Degiannis et al., one month after the end of hospitalization related to the treatment of non-iatrogenic limb artery injury, 95% of patients had a palpable pulse in the distal section in relation to the injury site. It was also observed that limb disability was more often associated with an injury which resulted in the coexistence of damage to the artery and peripheral nerves than in the case of isolated artery damage [6].

In a study conducted by Padayachy et al., in a retrospective evaluation lasting 5 years, 115 patients treated for a brachial artery injury were analyzed. Stab wounds were the most common cause of damage in this group of patients. Most of the injuries were treated with an inverted saphenous vein graft. The authors noticed that due to the good collateral circulation in the upper limb, most of these injuries do not require amputation, even when the treatment intervention is delayed [7]. In severe cases of brachial artery trauma (artery disruption, blood clot occluding the lumen of the artery, compression of the artery by a hematoma, bone fragment, or foreign body), the risk of amputation of part of or the entire damaged limb is real.

## 3. Limitation

The limitation of the presented case is the lack of imaging diagnostics showing the effect of the performed surgical treatment. Such diagnostics were abandoned due to the good clinical effect of the treatment, as well as the intention to avoid an additional dose of ionizing radiation and an additional dose of nephrotoxic contrast agents.

## 4. Conclusions

(1) The presented case emphasizes the importance of computed tomography angiography as a precise diagnostic tool facilitating the planning of a surgical procedure in patients with an occupational upper limb injury.
(2) In patients with extensive limb injury, CTA is useful for confirming and locating the source of vascular injury. In patients with suspected vascular injury, CTA may be the examination that determines the diagnosis.

## Data Availability

Not applicable.

## References

[1] Ball C.G., Rozycki G.S., Feliciano D.V. (2009). Upper extremity amputations after motor vehicle rollovers. J. Trauma.

[2] Liang N.L., Alarcon L.H., Jeyabalan G., Avgerinos E.D., Makaroun M.S., Chaer R.A. (2016). Contemporary outcomes of civilian lower extremity arterial trauma. J. Vasc. Surg..

[3] Kelly S.P., Rambau G., Tennent D.J., Osborn P.M. (2019). The Role of CT Angiography in Evaluating Lower Extremity Trauma: 157 Patient Case Series at a Military Treatment Facility. Military Med..

[4] Ergunes K., Yilik L., Ozsoyler I., Kestelli M., Ozbek C., Gurbuz A. (2006). Traumatic brachial artery injuries. Tex Heart Inst. J..

[5] Zellweger R., Hess F., Nicol A., Omoshoro-Jones J., Kahn D., Navsaria P. (2004). An analysis of 124 surgically managed brachial artery injuries. Am. J. Surg..

[6] Degiannis E., Levy R.D., Sliwa K., Potokar T., Saadia R. (1995). Penetrating injuries of the brachial artery. Injury.

[7] Padayachy V., Robbs J.V., Mulaudzi T.V., Pillay B., Paruk N., Moodley P. (2010). A retrospective review of brachial artery injuries and repairs—Is it still a “training artery”?. Injury.

